# Management of Refractory Post-operative Osteomyelitis and Discitis: A Case Report

**DOI:** 10.7759/cureus.52620

**Published:** 2024-01-20

**Authors:** Chase A DeLong, Malek Bashti, Long Di, Sumedh S Shah, Emade Jaman, Gregory W Basil

**Affiliations:** 1 Neurological Surgery, University of Miami Miller School of Medicine, Miami, USA

**Keywords:** extreme lateral interbody fusion, xlif, post-operative osteomyelitis, post-operative discitis, management, refractory discitis, refractory osteomyelitis

## Abstract

Vertebral osteomyelitis/discitis is a relatively rare disease but is a known potential complication of spinal surgical intervention. In general, the first-line treatment for this condition is targeted antibiotic therapy with surgical intervention only utilized in refractory cases with evidence of extensive damage, structural instability, or abscess formation. However, surgical best practices have not been established for osteomyelitis, including indications for anterior lateral interbody fusion (ALIF), posterior lateral interbody fusion (PLIF), or direct lateral interbody fusion (DLIF). This case provides a discussion of the indications that led to a direct lateral approach in the setting of refractory osteomyelitis/discitis, supporting factors that led to its success, and the efficacy of utilizing intraoperative neuromonitoring in cases of infection.

## Introduction

Osteomyelitis is a rare pathology, with an incidence of roughly 4.8 per 100,000 people in the United States [1]. Roughly 80% of cases of osteomyelitis occur post-operatively or post-traumatically, with *Staphylococcus aureus* (*S. aureus*) accounting for about 75% of chronic osteomyelitis cases [2]. Osteomyelitis of the spine is of particular concern given the possibility of infectious spread to the epidural space leading to potential motor deficits.

The first-line treatment for spinal osteomyelitis is typically targeted intravenous antibiotic therapy [3,4]. In refractory cases unresponsive to antibiotics, the standard of care procedures for surgical intervention have not yet been definitively decided. In general, surgical intervention is generally considered in a number of circumstances: 1) acute neurologic decline, 2) disease progression in spite of maximal medical management, and 3) progressive bony destruction or deformity [3-5]. Even after deciding to proceed with surgical intervention, there are still questions regarding the best approach: anterior vs posterior, single-stage vs two-stage, and whether instrumentation should be utilized [5]. In general, infectious disease experts have expressed concern regarding instrumentation in an active infection due to concern for biofilm formation [6,7]. There is, however, good literature on the safety and efficacy of titanium instrumentation in the context of active infection [6,8]. However, recent studies, such as a retrospective analysis by Dietz et al., showed decreased recurrence of infection, reoperation rates, and complications in patients who underwent surgical decompression and fusion compared to those who underwent decompression alone [9]. While there is discussion debating the anterior vs posterior surgical approach, there is a growing body of literature discussing the use of the direct lateral approach for the treatment of osteomyelitis. Of the studies available, the direct lateral approach has so far been demonstrated as an efficacious consideration for osteomyelitis [10,11]. However, given that there are no best practices in place regarding general surgical intervention for refractory osteomyelitis, the indications for direct lateral interbody fusion (DLIF) utilization in the scope of osteomyelitis are not established. The absence of such best practices adds to the difficult-to-manage post-operative nature of aggressive osteomyelitis and discitis in patients with multiple comorbidities. This case report highlights an example of such a scenario, emphasizing the importance of individualized patient care and the potential need for advanced surgical intervention in refractory cases.

## Case presentation

**Figure 1:**
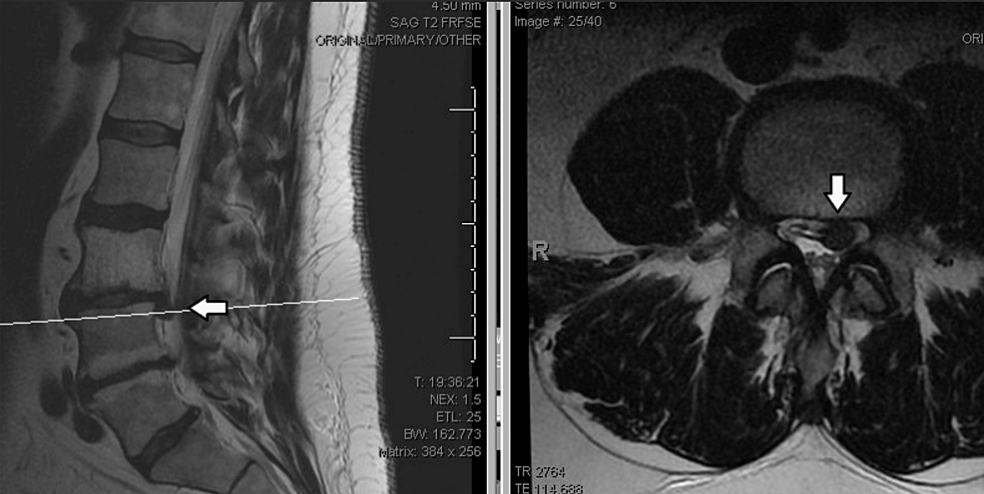
Pre-operative MRI Scan of Lumbar Spine, with Arrows Denoting L3/4 Disc Herniation Pre-operative MRI demonstrating multilevel spondylosis and a focal disc herniation at L3-4.

A 57-year-old male, with a history of endocarditis, poorly controlled diabetes mellitus, and obesity, presented with severe lower extremity radiculopathy. Initial imaging demonstrated multilevel spondylosis and a focal disc herniation at L3-4 (Figure 1). Despite undergoing a microdiscectomy a year prior, the patient experienced only temporary relief, with pain returning two weeks post-operation.

Given the absence of back pain and the persistence of severe radiculopathy, the patient underwent a revision L3-4 left microdiscectomy. Despite encountering intraoperative complications, such as an adherent dura necessitating additional lateral exposure, which left only a few millimeters of pars intact, the immediate postoperative period was uneventful, and the patient experienced complete relief of leg pain.

Immediately post-operation, the patient did very well and endorsed complete relief of his leg pain with no back pain. Post-operatively, he was started on intravenous (IV) vancomycin and ceftriaxone for 10 days. At two weeks post-operation, the patient presented with mild back pain, which paled compared to his pre-operative leg pain. However, his condition worsened over the subsequent two weeks, exhibiting severe back pain and limited mobility. Despite normal laboratory findings with slightly elevated ESR and CRP, a washout procedure 3.5 weeks post-operation revealed gross purulence, prompting immediate wound and disc space irrigation and the insertion of two drains. Cultures were positive for *Staphylococcus epidermidis* (*S. epidermidis*). The patient was started on IV cefepime and vancomycin while admitted and was transitioned to oral linezolid and rifampin for two weeks, followed by oral cephalexin and rifampin for 10 weeks. These durations had the potential for extension until lab values and imaging demonstrated resolution of the infection.

**Figure 2:**
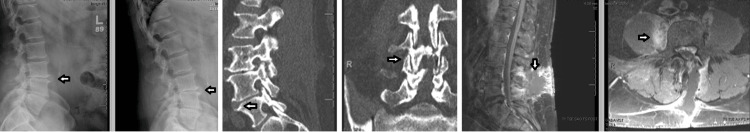
Lumbar XR and MRI with Arrows Denoting Psoas Abscess Imaging obtained after decompression and subsequent washout procedure, demonstrating psoas abscess. Demonstrates (A) neutral and (B) extension lumbar XR, (C) lateral + (D) AP lumbar CT, and (E) lateral + (F) AP MR imaging w/o contrast. XR = x-ray, AP = anterior-posterior, MR = magnetic resonance

Despite the washout and antimicrobial therapy, the patient's condition continued to deteriorate, exhibiting severe back and right thigh pain with imaging, suggestive of a progressive right-sided psoas abscess. Subsequent aspiration of the abscess seven weeks post-operation grew *S. epidermidis* (Figure 2).

Management and outcome

**Figure 3:**
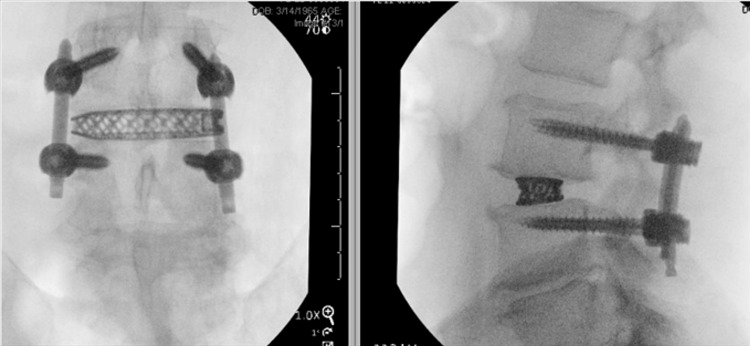
Lumbar XR with Post-operative Instrumentation Post-operative imaging demonstrating instrumentation placed via aggressive discectomy and DLIF for source control and stabilization at L3-4.

Three management strategies were considered: a second washout, transfer to rehabilitation with continued IV antibiotics, or fusion surgery. The decision was made to perform a fusion procedure with re-washout of the surgical bed to achieve source control and stabilization via an aggressive discectomy and lateral interbody cage. A posterior approach was not chosen due to our belief that we would not be able to sufficiently clean the disc space through a small posterior corridor. Intra-operatively, the right psoas abscess was not encountered through the surgical corridor. Antibiotic-impregnated irrigation was used to wash the approach window and the L3-4 disc space prior to lateral instrumentation (Figure 3). The operation utilized electromyogram (EMG) intraoperative nerve monitoring (IONM) to measure spontaneous electromyogram activity and reduce the risk of iatrogenic neuropathic damage to the lumbar plexus. Post-operatively, an MRI lumbar spine MRI was obtained to further localize the abscess, and the patient was taken to the IR suite for aspiration and drain placement. Following the DLIF and aspiration, the patient’s back pain immediately improved post-operation. He was discharged two days post-operation with IV daptomycin for eight weeks and oral rifampin for 12 weeks. At the two-week and six-week follow-ups, the patient reported complete symptomatic relief with no recurrence of back or radicular leg pain. He only endorsed very mild, gradually improving right leg discomfort when lifting his leg and fully flexing his hip. Follow-up CT lumbar imaging was obtained four weeks post-operation, which demonstrated seated and intact hardware without any signs of recurrent infection.

## Discussion

Table 1 demonstrates a summary of our literature review for the use of XLIF for the treatment of spondylodiscitis, with the authors, title, source, purpose, and findings of each paper listed.

**Table 1:** Literature Review of XLIF/LLIF Being Utilized for the Treatment of Osteomyelitis/Discitis (Spondylodiscitis) XLIF: extreme lateral interbody fusion, LLIF: lateral lumbar interbody fusion, ALIF: anterior lumbar interbody fusion, PLIF: posterior lumbar interbody fusion, ODI: Oswestry disability index, VAS: visual analogue scale

Authors	Title	Source	Purpose	Findings
Blizzard et al. [10]	Extreme Lateral Interbody Fusion with Posterior Instrumentation for Spondylodiscitis	Journal of Clinical Neuroscience	Case series of 11 spondylodiscitis patients treated with XLIF	XLIF is a useful approach that offers an alternative to the challenges with a traditional anterior and/or posterior approach.
Tani et al. [12]	A New Treatment Algorithm That Incorporates Minimally Invasive Surgery for Pyogenic Spondylodiscitis in the Thoracic and Lumbar Spines: The Results of Its Clinical Application to a Series of 34 Patients	Medicina	Case series of 34 patients treated for pyogenic spondylodiscitis following an algorithm developed at Kansai Medical University	While the algorithm is a step towards best-practice principles, it currently risks oversimplification and removes important aspects of the decision-making process for serious infection.
Karikari et al. [11]	Extreme Lateral Interbody Fusion Approach for Isolated Thoracic and Thoracolumbar Spine Diseases: Initial Clinical Experience and Early Outcomes	Journal of Spinal Disorders & Techniques	To describe the initial clinical experience and outcomes with the extreme lateral antibody fusion (XLIF) approach for spinal diseases requiring access to the thoracic cavity	One patient in the case series was treated for osteomyelitis/discitis, and died 3 months after the procedure due to metastatic breast cancer. The case series demonstrated XLIF as a useful approach for thoracic spinal disease management, especially in the elderly and those with significant comorbidities.
Yang et al. [13]	Minimally Invasive Lateral Lumbar Intervertebral Fusion Versus Traditional Anterior Approach for Localized Lumbar Tuberculosis: A Matched-Pair Case-Control Study	The Spine Journal	Case-control study comparing patients with lumbar tuberculosis treated with LLIF vs ALIF	LLIF demonstrated shorter follow-up time, incision length, operation time, and blood loss. Overall LLIF was demonstrated as an effective option for lumbar tuberculosis, with notable benefits over other methods.
Raymaekers et al. [14]	Extreme Lateral Interbody Fusion as a Feasible Treatment for Thoracolumbar Spondylodiscitis: A Multicenter Belgian Case-Series	World Neurosurgery	Case series of 7 patients who underwent XLIF for spondylodiscitis	XLIF with add-on percutaneous pedicle screw fixation is a potentially feasible, safe, and valuable choice for spondylodiscitis treatment.
Timothy et al. [15]	Extreme Lateral Interbody Fusion (XLIF) as a Treatment for Acute Spondylodiscitis: Leeds Spinal Unit Experience	Journal of Clinical Neuroscience	Cohort study of patients with spondylodiscitis treated with XLIF	XLIF with debridement was demonstrated as an effective treatment for spondylodiscitis, with ODI and VAS improvement.
Pojskić et al. [16]	Extreme Lateral Interbody Fusion (XLIF) in a Consecutive Series of 72 Patients	Bosnian Journal of Basic Medical Sciences	Evaluate the safety and efficacy of XLIF in spinal canal stenosis and spondylodiscitis via retrospective analysis	XLIF with supplemented instrumentation is a safe treatment for spondylodiscitis, but indications should be considered carefully.

Post-operative discitis and osteomyelitis present formidable management challenges due to their complex nature and resistance to conventional treatment strategies [17]. Antimicrobial therapy, washout procedures, and surgical interventions are usually the standard treatments. The decision to choose a particular surgical approach remains a contentious topic that requires continued exploration.

One surgical strategy, spinal fusion, has shown promising results in refractory cases. The procedure's primary purpose is to stabilize the spinal column, resulting in reduced pain and improved patient mobility. Recently, there has been a significant body of literature to support the use of titanium hardware in active infections due to its biocompatibility and resistance to biofilm formation infection. Literature support for this is seen in the 2018 study by Mok et al., where titanium cages were successfully employed in spondylodiscitis management, leading to significant functional improvement and pain reduction [18]. Further reviews have demonstrated *S. aureus* is less adherent to solid titanium than titanium alloys, stainless steel, or polyethyletherketone (PEEK), with the texture of the hardware (smooth vs porous) having no significant impact [6,8].

There is, however, some discordance on the need for long-term suppressive antibiotic therapy in patients who receive instrumentation in the context of active local infection. While our practice pattern has been to discontinue all antibiotic therapy assuming resolution of all laboratory markers of infection, and radiographic evidence of infection, some infectious disease experts advocate for lifelong antibiotic therapy [19,20]. This disagreement can have substantial implications for patients and requires a multi-disciplinary agreement. Again, given the significant body of literature demonstrating the safety of titanium instrumentation in these cases, we would advocate for a more pragmatic approach.

Osteomyelitis primarily impacts the anterior and middle columns of the lumbar spine, leaving the posterior elements mostly untouched [3,17]. This situation makes the lateral transpsoas approach, such as the direct lateral interbody fusion (DLIF), an optimal treatment choice as it offers a direct surgical pathway to the vertebral body without disrupting the unaffected posterior structures or risking damage to the neural elements [21,22]. Moreover, it obviates the need for mobilization of the great vessels, which is typically required in the conventional anterior approach. In addition to its specific suitability for osteomyelitis, the lateral transpsoas approach offers general benefits, such as reduced tissue trauma, decreased blood loss, reduced traction on neural elements, and shorter operative time [23,24]. In this particular case, the transpsoas approach was especially attractive given the concomitant psoas abscess, which could be directly washed out via this approach. These attributes are especially beneficial when treating patients with osteomyelitis, who often present in less-than-ideal medical conditions [17,25].

A review of the literature demonstrated findings that overall support DLIF as a means of treating osteomyelitis/discitis, with an emphasis on the current uncertainty around the indications for such an approach. Karikari et al. demonstrated the DLIF approach as a less invasive alternative to traditional open procedures, especially in elderly patients or those with multiple comorbidities [11]. While an algorithmic approach to patient care was proposed by Tani et al. for determining when to use DLIF, the authors cautioned that this approach may oversimplify the management of complex refractory osteomyelitis cases [12]. Comparisons between LLIF and ALIF/PLIF for treating lumbar infections showed substantial advantages associated with LLIF, including shorter follow-up time, smaller incision length, reduced operation time, and less blood loss [13]. While other studies also demonstrated similar benefits, Pojskić et al. showed risks with these benefits, primarily a higher rate of worsening neurological deficits post-operation. The most commonly noted deficit was ipsilateral thigh weakness, most frequent in spondylodiscitis patients, which the authors attributed to paravertebral muscle infection, rather than due to the DLIF approach itself. The authors also noted a higher rate of non-fusion when utilizing the DLIF approach, also attributed to infection, as well as shorter follow-up [16]. These collective findings underscore the need for formal guidelines for employing the DLIF approach in refractory osteomyelitis/discitis cases.

One of the major concerns with a lateral approach is of course the lumbar plexus. While we did use evoked EMG during the surgery, there is some question as to the reliability of this neuromonitoring in the context of an active infection in the psoas muscle. While our case did not result in any neurologic deficit, we cannot merely extrapolate our results to other patients without further investigation. In general, there is a good body of literature discussing the occurrence of plexopathy following a direct lateral approach [26,27]. There is also literature to suggest that retractor time may be related to the rate of postoperative neural complications [28,29]. However, infection may pose a unique challenge. Given the edematous psoas muscle, and likely co-existing irritation of the plexus (as evidenced by our patient’s anterior thigh pain), it is possible that these neural structures are even more sensitive to neuropraxia. Additionally, it is unclear whether the presence of an abscess/phlegmon could potentially alter the efficacy of evoked EMG testing. Given the uncertainty of this monitoring technique in the context of abscess infection, there is some literature to suggest supplementation with neurophysiologist-controlled testing. Indeed, a study by Riley et al. demonstrated the lowest rate of motor or sensory neurologic deficit, both post-operative and 12 months after surgery, for neurophysiologist-controlled T-EMG monitoring supplemented with MEP monitoring (NC-MEP) compared to surgeon-directed T-EMG (SD-EMG) and neurophysiologist-controlled T-EMG (NC-EMG) [30]. This significant difference in outcome based on IONM modality demonstrates that IONM is subject to notable efficacious variability, and further studies should be performed to identify such influential factors.

Another concern in the timely response to refractory discitis is the potential sequelae. Untreated discitis leads to further inflammation of the disc and contiguous spread to adjacent vertebrae and can even result in neurological dysfunction [31]. While not detailed in the literature, it is reasonable to conclude that inflammation and destruction caused by discitis may increase the risk of disc herniation. While herniation most frequently presents with symptoms not raising concern for a medical emergency (pain, sensory loss, or muscle weakness in a radicular pattern), the potential for complications such as cauda equina syndrome (CES) increases the urgency of treating refractory discitis [32]. Evaluation by Bečulić et al. demonstrated CES patients as having chronic radicular pain 70% of the time, with 30% of patients having new onset symptoms. The analysis further demonstrated worse post-operative outcomes when there was a longer period between symptom presentation and surgical intervention for CES, with 48 hours being an optimal window [33]. While the most common treatment for discitis is antibiotics without surgical intervention, refractory disease warrants consideration of surgical debridement, given the significant risks of permanent dysfunction, such as in the setting of emergent CES [31].

This case underscores the potential efficacy of lateral spinal fusion surgery, complemented by intraoperative neuromonitoring, in managing refractory postoperative osteomyelitis and discitis. The case presented herein serves mainly as an example case by which to discuss the importance of a more nuanced understanding of neuromonitoring in this particular context. It additionally serves to raise the question of long-term suppressive antibiotic therapy in patients who receive instrumentation with active infection. These findings warrant further research to better understand the mechanisms behind the successful outcomes and their potential for broader application in similar cases. Moreover, it calls for the development of specific guidelines regarding patient selection, surgical approach, and postoperative care for managing such complex cases. Future studies should also delve deeper into the reliability and nuances of neuromonitoring in infected cases, given its instrumental role in this case's successful outcome. The importance of further studies evaluating IONM for osteomyelitis cases is highlighted by over 15% of osteomyelitis patients suffering from permanent neurological damage, providing the opportunity for IONM to prevent avoidable iatrogenic contributions to an already high rate of neurological deficits [34]. There is an opportunity for even greater insight into IONM with osteomyelitis; as in this case, the most pertinent factor is the efficacy of IONM in nerve plexuses with active infection. Our literature review demonstrated no such findings at this time.

## Conclusions

The case emphasizes the need for individualized treatment strategies in the management of refractory post-operative osteomyelitis and discitis. In such scenarios, aggressive surgical intervention, such as fusion surgery, may be required for disease control and to improve patient outcomes. The main takeaway is not that spinal fusion should be the first line of treatment, but rather in patients with lumbar discitis unresponsive to systemic antibiotic therapy and showing disease progression despite conservative measures, a lateral approach may allow for proper source control and spine stabilization, facilitating healing. Future research and clinical practice should focus on identifying such patients earlier in their disease course to provide tailored, effective care.
